# 680. Outpatient Burn Therapy Delivery: Patient Needs versus Payer Limitations

**DOI:** 10.1093/jbcr/irag033.561

**Published:** 2026-04-06

**Authors:** Derek O Murray, Audrey M O'Neil, Karen J Richey, Kevin N Foster

**Affiliations:** Diane & Bruce Halle Arizona Burn Center - Valleywise Health, Phoenix, AZ; Richard M Fairbanks Burn Center, Indianapolis, IN; Diane & Bruce Halle Arizona Burn Center - Valleywise Health, Phoenix, AZ; Diane & Bruce Halle Arizona Burn Center - Valleywise Health, Phoenix, AZ

## Abstract

**Introduction:**

Few studies have examined outpatient burn therapy delivery, as much of this care is managed by rehabilitation providers outside of burn centers. Long term burn injury sequela are not recognized as chronic conditions, and third-party payers may limit support for long-term rehabilitation needs of burn survivors. The purpose of this study is to explore the impact of payer type on key elements of outpatient therapy delivery.

**Methods:**

Data was analyzed from a dedicated burn outpatient therapy department embedded within a verified burn center and was analyzed over an 11 month period, October 2024 to August 2025. Collected variables included patient insurer, therapy procedures delivered, duration of therapies, procedures per session, total treatments, and whether a patient was denied additional therapy services prior to therapy goals being attained. Patients (n = 32) were divided into two groups based on category of insurer: collaborative (Medicare, Worker’s Compensation), and constraining (Managed Care, Medicaid, Medicare HMO, Commercial payers). Self-pay patients were excluded from the analysis. Student’s t-test was utilized to determine statistical differences between the two groups.

**Results:**

Significant differences were observed in provision of therapy services between the collaborative (n = 9) and the constraining payers (n = 23). Duration of therapy treatment averaged (152.8 vs 52.2 days, *p*=.0248), and denial or exhaustion of therapy benefits before patient goals were met occurred in (0% of the collaborative group vs 43.5% of the constraining group, (*p*<.001). Average total treatment differences between the groups approached significance, (26.5 versus 12.2 sessions, *p*=.0580). No significant differences were found in average procedures (135.1 vs 69.7; *p*=.109) or procedures per session, (4.51 vs 5.3 *p*=.248).

**Conclusions:**

Outpatient burn therapy delivery is constrained by third-party payer policies rather than patient needs, potentially limiting functional recovery and quality of life. These disparities highlight the need for advocacy to align insurance coverage with evidence based. Further research and advocacy is required to match needs of burn survivors to therapy supported by payers and insurance regulators.

**Applicability of Research to Practice:**

Outpatient therapists should anticipate variability in payer support, and prepare care strategies accordingly to optimize outcomes for burn survivors.

**Funding for the study:**

N/A.

